# Advances in Alpha Herpes Viruses Vaccines for Human

**DOI:** 10.3390/vaccines11061094

**Published:** 2023-06-12

**Authors:** Madalina Preda, Loredana Sabina Cornelia Manolescu, Razvan Daniel Chivu

**Affiliations:** 1 Department of Microbiology, Parasitology and Virology, Faculty of Midwives and Nursing, “Carol Davila” University of Medicine and Pharmacy, 020021 Bucharest, Romania; 2 Research Department, Marius Nasta Institute of Pneumology, 050159 Bucharest, Romania; 3 Department of Virology, Institute of Virology “Stefan S. Nicolau”, 030304 Bucharest, Romania; 4 Department of Public Health and Health Management, Faculty of Midwifery and Nursing, “Carol Davila” University of Medicine and Pharmacy, 050474 Bucharest, Romania; razvan.chivu@umfcd.ro

**Keywords:** HSV, VZV, immunity, vaccine

## Abstract

Alpha herpes simplex viruses are an important public health problem affecting all age groups. It can produce from common cold sores and chicken pox to severe conditions like encephalitis or newborn mortality. Although all three subtypes of alpha herpes viruses have a similar structure, the produced pathology differs, and at the same time, the available prevention measures, such as vaccination. While there is an available and efficient vaccine for the varicella-zoster virus, for herpes simplex virus 1 and 2, after multiple approaches from trivalent subunit vaccine to next-generation live-attenuated virus vaccines and bioinformatic studies, there is still no vaccine available. Although there are multiple failed approaches in present studies, there are also a few promising attempts; for example, the trivalent vaccine containing herpes simplex virus type 2 (HSV-2) glycoproteins C, D, and E (gC2, gD2, gE2) produced in baculovirus was able to protect guinea pigs against vaginal infection and proved to cross-protect against HSV-1. Another promising vaccine is the multivalent DNA vaccine, SL-V20, tested in a mouse model, which lowered the clinical signs of infection and produced efficient viral eradication against vaginal HSV-2. Promising approaches have emerged after the COVID-19 pandemic, and a possible nucleoside-modified mRNA vaccine could be the next step. All the approaches until now have not led to a successful vaccine that could be easy to administer and, at the same time, offer antibodies for a long period.

## 1. Introduction

**Figure 1:**
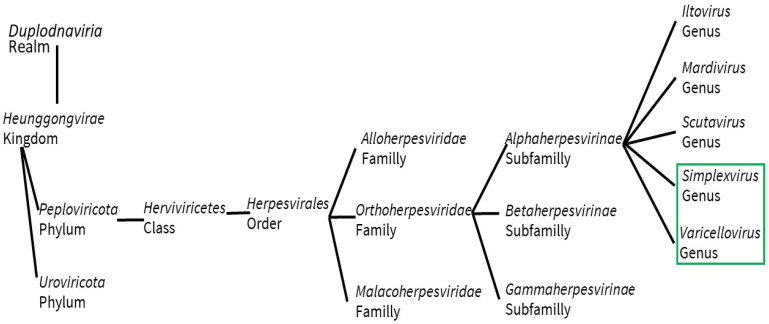
Simplexvirus and Varicellovirus Genera taxonomic inclusion, according to the International Committee on Taxonomy of Viruses. Underlined in the green box are the discussed viruses.

According to the International Committee on Taxonomy of Viruses, orthoherpesviridae family is divided into three sub-families alphaherpesvirinae, betaherpesvirinae, and gammaherpesvirinae [1]. The two human pathogenic subtypes (herpes simplex virus type 1, HSV-1; and herpes simplex virus type 2, HSV-2) [2,3]; and varicella-zoster virus (VZV) belong to the alpha subfamily (Figure 1). 

In 2016, 491.5 million people between 15 and 49 were reported globally to have HSV type 2 infection [4]. Regarding gender, women are more affected, with 313.5 million HSV-infected women compared with 178.0 million men [4]. After the Western Pacific, South-East Asia, and Americas Regions, the WHO African Region had the highest infection rate (102.9 million females and 59.3 million men) [4]. The estimated global prevalence of HSV type 2 in the population of 3735.6 million persons aged 15 to 49 was 13.2%, with the majority being highest in the populations of Africa, the Americas, and among women [4]. For both HSV-1 and -2, virus shedding can be symptomatic or asymptomatic [5]. This might be one of the characteristics which contributes to a high global frequency since contagious infected persons can be asymptomatic and unintentionally spread the virus [5]. HSV-1 can infect vaginal mucosal epithelial cells, although it usually infects orofacial mucosal epithelial cells, where productive infection occurs [2]. The HSV-2 disease is a widespread sexually transmitted illness that is also the most common cause of focused, sporadic encephalitis in the United States and a significant contributor to infectious blindness in the West [1]. Additionally, it contributes significantly to newborn mortality [2]. 

Without considering the price of preventing or treating neonatal herpes, the present value of lifetime direct medical expenditures for genital herpes was $972 per treated case or $165 per initial diagnosis of genital herpes infection (in 2019 USD) [6]. 27% of all expenses were related to the initial clinical appointment where genital herpes was identified [6]. Additional clinical visits and drugs for genital herpes accounted for 13% and 60% of total lifetime expenses, respectively [6].

Varicella (chickenpox) is a highly contagious systemic illness with a rapid onset. It occurs due infection with the varicella-zoster virus a DNA virus that belongs to the herpesvirus family [7]. After the first infection, VZV remains dormant in the body (in the sensory nerve ganglia) [7]. Herpes zoster is brought on by the latent reactivation of the VZV (shingles) [7].

Varicella is a frequent illness among children in temperate regions, with most cases occurring in the winter and spring [8]. It causes more than 9000 hospital admissions per year in the US. The age interval between 4 to 10 presents the highest incidence [8]. The prevalence of varicella infection is 90% [8]. 

Varicella infections and its consequences have significantly decreased since the varicella vaccination campaign was launched in 1995 [8]. It is advised that children receive routine immunizations [8]. Three days after exposure, immunization may still help children have better outcomes [8].

Although they belong to the same subfamily, these three viruses have similarities in structure, first infection, latency, and reactivation. Still, the pathology and immune response produced varies, especially concerning VZV’s available vaccine [9].

Despite extensive research, the optimum vaccine candidate for controlling HSV illness should be improved [10]. Modern vaccination strategies may better boost protective host immunity [10].

We conducted a comprehensive literature search on herpes simplex virus infection and vaccine approaches using PubMed, Google Scholar, National Library of Medicine, Web of Science, and Scopus (Elsevier) databases and official websites of WHO and CDC to identify relevant data for inclusion in our review. The major research terms were “Herpes simplex virus”, “HSV”, “HSV infection”, “HSV vaccine”, “HSV immunization”. Inclusion criteria were established based on Published in a peer-reviewed journal written in English or French, full text available, examination of the safety or efficacy of a vaccine against herpes simplex virus (HSV), the use of a randomized controlled trial (RCT) design, or a well-designed observational study with a large sample size and appropriate controls, being a thoroughly performed review, reporting clinical outcomes such as reduction in HSV shedding or symptoms, or induction of neutralizing antibodies, examining the effect of a specific intervention or treatment. Exclusion criteria were based on not being published in a peer-reviewed journal, written in a language other than English or French, using a study design that is poorly controlled or has a small sample size, published more than 30 years ago. Full-text articles were assessed for eligibility, with final inclusion decisions made by consensus. 

## 2. Structure, Pathology, and Immune Response

### 2.1. Structure

Most genes and related mechanisms are shared by the three alpha herpes viruses at the molecular level [9]. Moreover, they have a similar electron microscopic toroid structure [11,12]. Given these findings, it may come as a surprise that HSV and VZV cause pathogenicity in quite distinct ways in the human host [9] and that vaccines are not available for herpes viruses 1 and 2. Each virus has acquired a specific pattern of infection and particular skills to thwart the host’s innate and adaptive immunity [9].

Herpesvirus (HSV and VZV) proteomes are highly similar [9]. Simplexvirus and Varicellovirus Genera establish a latent infection in the sensory ganglia that innervate the site of infection in the periphery [1]. All three viruses (HSV-1, HSV-2, and varicella) share many genes and have similar molecular activities [1]. The HSV genome is larger (~152 Kbp) than that of VZV (~125 Kbp), and while HSV can encode six immediate-early proteins, VZV encodes only three [1]. Some of these proteins are also involved in eluding the immune response. HSV has twelve short unique regions, while VZV only has four [1]. Given these facts, it could come as a surprise that HSV and VZV use such divergent approaches in human pathogenesis [1]. Each virus has its own distinct mode of infection and has refined strategies to evade the body’s natural defenses [1]. Although they have similar characteristics, some of the differences between HSV and VZV may also be the answer to the delay in obtaining a vaccine against herpes simplex virus [9]. The key may be the 41 “core” proteins common to all herpes viruses that code for fundamentally important processes, such as the essential proteins for DNA replication, capsid elements, and a few enzymes [9,13]. 

#### 2.1.1. Herpes Simplex Viruses (HSV)

**Figure 2:**
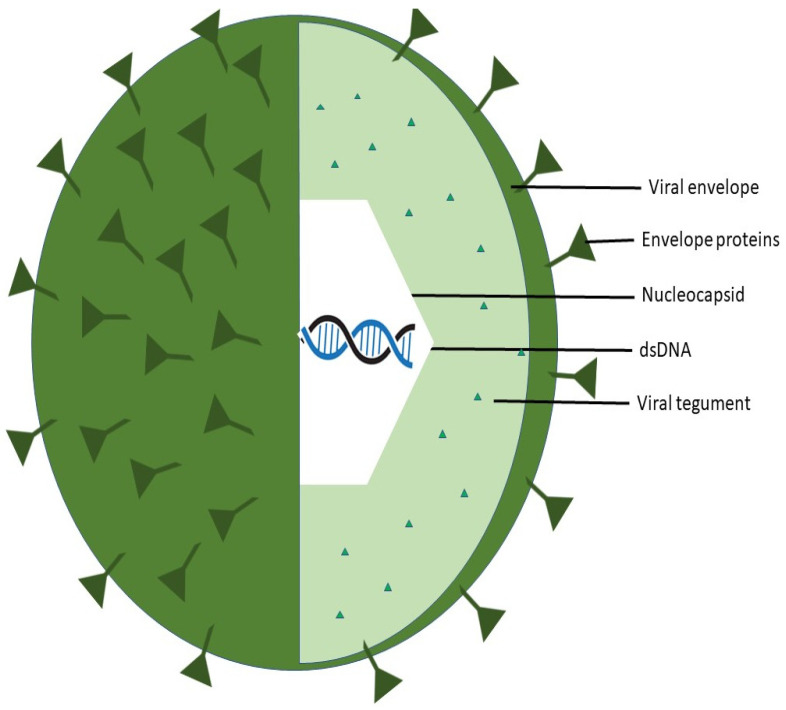
Herpes Simplex Virus structure (adapted after [1]).

Herpesviruses have a distinctive four-layered structure: an icosapentahedral capsid made of capsomers encloses the core containing the large, double-stranded DNA genome [14]. The tegument, an amorphous protein coat, envelops the capsid. It is enclosed in a lipid bilayer coat that contains glycoproteins (Figure 2) [14]. 

**Figure 3:**
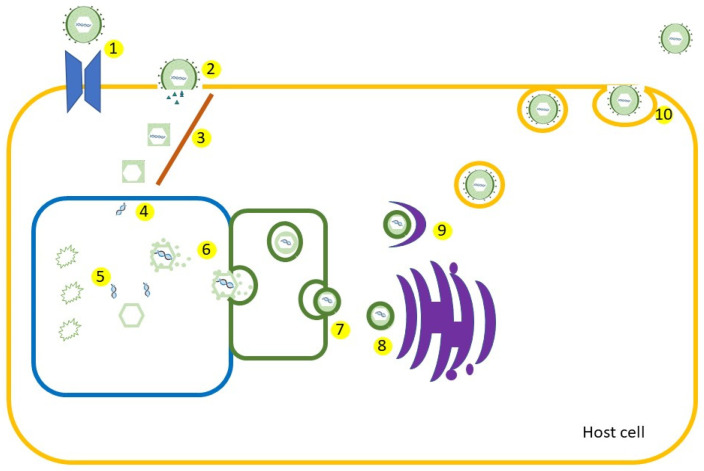
HSV replication. 1—attachment to the host cell; 2—entry in the cell; 3—microtubule transfer to nucleus; 4—viral DNA enters the nucleus of the cell; 5—viral genome is replicated; 6—tegument is formed and attached; 7—nuclear egress; 8—tegument layer is added to the capsid; 9—through Golgi apparatus and endosomes a protein-studded envelope is added; 10—exocytosis (Adapted after [21,22]). Colors and shapes are schematic, not according to microscopic appearance.

The double-stranded DNA herpesviruses can have genomes as long as 240 kbp [15]. A complex T = 16 icosahedral capsid with a diameter of 1250 Å contains the viral DNA [15]. The proteinaceous tegument, which is encircled by a lipid envelope generated from the host, contains the DNA-containing capsid, or nucleocapsid [15]. Glycoproteins that facilitate viral attachment and entry are embedded throughout the viral envelope [15]. Four viral envelope glycoproteins (gB, gD, and the heterodimer gH/gL) and a cell surface gD receptor are necessary for HSV entrance into cells [16]. HSV virions infiltrate host cells by fusing their envelopes with the host cell’s plasma membrane, allowing the nucleocapsid and tegument to enter the cytoplasm [15] (Figure 3).

Glycoprotein B (gB) and gC adhere first to the plasma membrane by binding to glycosaminoglycans (GAG) [16]. After binding to GAGs, gD interacts with several entrance receptors, including 3-O-sulfated HS, nectin-1 and -2, and the herpesvirus entry mediator [16]. The interactions between gB and paired immunoglobulin-like type 2 receptor, myelin-associated glycoprotein, and non-muscle myosin IIA have also been shown to have a role in HSV entrance, according to several investigations [16]. HSV-1 enters by endocytosis by interacting with certain integrins via the gH/gL proteins [16]. Receptor expression varies between tissues and cell types, which affects viral tropism [16]. 

It was noticed that on the surface of virions and infected cells, heterodimer of glycoproteins E (gE) and I (gI) can be expressed [17]. The virus spreads from cell to cell through the gE/gI heterodimer [17]. Importantly, glycoprotein E also helps the virus evade the immune system [17]. Additionally, it has been demonstrated that gE attaches to the immunoglobulin G (IgG) Fc domain and blocks the immunologic functions that the IgG Fc domain facilitates through a mechanism known as antibody bipolar bridging [17]. It was found that the virus can be shielded and protected from Fc-mediated immunological responses, such as viral neutralization and antibody-dependent cellular cytotoxicity, by this binding capability [17]. Antibody and CD4+ T cell-dependent immunological responses to mgG2 have been documented [18]. Based on these results, mgG2 was proposed as a potential vaccine antigen against HSV-2 infection and illness [18].

By fusing with the plasma membrane of host cells, the nucleocapsid, and tegument of HSV virions gain access to the cytoplasm [19]. From the microtubule-organizing center, the nucleocapsid moves via microtubules to the nucleus [19]. The nucleocapsid docks with the nuclear pore complex before injecting the genome into the nucleus [5]. DNA leaves the capsid through a portal-vertex located on the axis of 5-fold symmetry in an icosahedral shape [15]. In addition, the portal vertex in the nucleus is responsible for packing the viral DNA into capsids [15]. Virion morphogenesis (VP5) begins in the nucleus with the construction of the procapsid, an icosahedral symmetric spherical shell composed of capsomeres made up of hexamers and pentamers of the major capsid protein pUL19 [20]. Procapsid assembly, which is nucleated around the dodecameric portal formed by pUL6, is promoted by the scaffold protein pUL26 and the heterotrimers of pUL38 (VP19C) and pUL18 (VP23), known as triplexes [20]. Procapsid maturation, which involves the angularization and ejection of the scaffold protein, happens when the viral DNA is forced into the shell [20]. After the viral genome has been replicated into a concatemer, the terminase complex (pUL33, pUL28, and pUL15) and the portal protein (pUL6) bundle the concatemer’s unit-length genomes into procapsids [19].

Lytic or latent replication is brought on by HSV infection [23]. During latency there is reduced gene expression and no viral particle generation. Coordinated gene expression during lytic replication creates infectious viruses [23]. The viral genome can reactivate, which creates infectious virions in response to the right stimuli [10]. Following entrance via a mucosal surface or injured skin, the intracellular replication begins at the original site of exposure, where it causes the primary infection in hosts [10]. Viral invasion into the peripheral nervous system can occur through nerve terminals in infected skin or mucosal tissue [10]. Axons are used for the retrograde movement of viral particles, and the virus’s genetic material is released into the nucleus [10]. The sensory ganglia must undergo this process to achieve a latent state until viral reactivation [10]. In the event of reinfection, freshly produced virions move anterogradely from the ganglia toward the nerve ends of innervated mucocutaneous locations (dermis or innervated tissue) [10]. In neurons, HSV establishes latency [23]. Although a recent study revealed that some non-neuronal cells in vitro may be also involved in latency, infection of susceptible non-neuronal cells usually results in lytic multiplication [23]. 

#### 2.1.2. Varicella Zoster Virus (VZV)

The icosahedral capsid shell of the varicella-zoster virus, composed of many viral proteins, encloses the highly structured dsDNA genome, shielding it from extracellular elements and immunological sensors in the cytoplasm [24]. 

The structure of the virus is related to vaccine development. Thus live attenuated VZV imitates an actual infection in order to mount a complete B and T cell response capable of attacking all of the almost 100 proteins presented by VZV [25]. This encompasses all of the viral particle’s constituent parts, including all of the glycoproteins that are not only visible on the surface of infected cells but also make up the viral particle itself [25]. The subunit vaccine, which is based entirely on the viral glycoprotein gE, can induce a B and T cell response against this protein that even outperforms the live attenuated vaccine [25].

During replication, after cell entry, the genomic DNA is released for replication in the viral nuclear factory once the virus has entered the cell and is carried by molecular motors along the microtubule system into the nucleus [24]. The freshly created viral DNA is crammed into the developing capsid shell, which is later evacuated from the nuclear membrane’s inner lamella by egress [24]. The VZV progeny virion matures at the plasma membrane, after which it buds at the cell surface, a highly unusual feature for herpesviruses [24]. Cell-cell fusion is easily facilitated to aid virus transmission via syncytia formation because the viral fusogenic glycoproteins are produced on the viral envelope and the membrane of infected cells [24].

Innate and adaptive host responses are important in the immune regulation of productive VZV infection [26]. The innate host immune response is an early host response that comprises natural killer (NK) cells, NK-T cells, type I (and) and type II (IFN), and is meant to block or limit viral transmission within the host [26]. The production of type I IFNs, which generates many IFN-stimulated genes and results in an antiviral state, is one of the signal transduction pathways engaged by the innate responses to virus infection [26]. These genes produce the Mx proteins, 2-5 oligoadenylate synthetase (2-5 OAS), and protein kinase R, which inhibit viral transcription, translation, and probably other viral activities, including viral DNA replication and assembly to cause an antiviral response [26]. Numerous viruses have developed mechanisms that hinder IFN production or obstruct the antiviral effects of IFNs [26].

CD3+ T cells, which include the CD4+, CD8+, and dual CD4+CD8+ T cell subpopulations, may reproduce infectious viruses and release them [27]. Transmission of VZV from respiratory epithelial cells to T cells occurs efficiently, most likely within the tonsils and other lymphoid organs that make up the Waldeyer’s ring, just as Epstein-Barr virus spreads from tonsil B cells to T cells [27]. VZV can also infect dendritic cells, which could aid in lymph node spread [27]. CD4+ T cells infected with VZV exhibit activation markers and skin-homing proteins such as cutaneous leukocyte antigen (CLA) and CC-chemokine receptor 4 (CCR4). The increased likelihood of their penetration into the skin and other tissues is due to this fact [27]. Additionally, VZV is able to cause naïve T cells to express skin-homing and activation proteins [27].

Varicella develops by T-cell infection, which spreads the virus to the skin and causes it to develop latency in the sensory nerve ganglia [28]. The polykaryocytes that produce VZV skin lesions are created by cell fusion and release a high amount of virus [28]. VZV infection, in contrast, does not cause T-cell fusion, enabling these cells to spread the virus to the skin and neurons [28]. The crippling disease postherpetic neuralgia and zoster are brought on by VZV reactivation from latency [28]. 

Infected keratinocytes release freshly formed virions from a lesion where VZV has suppressed the innate immune system [26]. Someviral proteins can prevent infected cells from responding to IFN: IFN production is prevented by IE62’s inhibition of TANK-binding kinase 1’s (TBK-1’s) phosphorylation of IFN regulatory factor 3 (IRF3), ORF47 kinase’s reduction of IRF3’s phosphorylation, and IE63’s inhibition of the phosphorylation of the eukaryotic initiation factor 2 and its downstream IFN effects [26]. The ORF66 kinase prevents STAT1, activated in response to IFN and IFN signaling, and upregulates IFN-stimulated genes from being activated (ISGs) [26]. By halting the breakdown of the NF-B inhibitor (IB), VZV also sequesters the p50-p65 heterodimer, the most prevalent form of NF-B, in the cytoplasm of epidermal cells [26]. This last impact is cell type-specific since VZV causes NF-B to be induced in monocytes [26].

### 2.2. Pathology

HSV-1 and HSV-2 infections can have asymptomatic, mild, or life-threatening effects [16]. Most immunocompetent people who get HSV develop a minor, self-limiting illness [16]. Cold sores, genital herpes, herpes stromal keratitis, eczema herpeticum, disseminated disease in the newborn, meningitis, and herpes simplex encephalitis are among the illnesses HSV may bring [16]. The severity of the condition is influenced by multiple characteristics, among which the microbiome could be mentioned [29]; for example, in a study on mice, the ones with antibiotics treatment and dysbiosis were more predisposed to HSV infection than the healthy controls [30].

Although HSV-1 is most commonly linked to orofacial lesions, it is also the most common cause of viral encephalitis in adults and the top infectious cause of blindness in industrialized nations [31].

The infection is persistent and is regularly characterized by reactivations at the infection site [4]. Orolabial herpes is frequently brought on by HSV type 1, which is typically spread through oral-to-oral contact (cold sores) [4]. In addition to encephalitis, type 1 herpes virus causes more frequently uncommon illnesses, such as keratitis and other ocular sequelae [4].

HSV-2 is mainly linked to newborn encephalitis and genital lesions [31,32,33], even though initial genital infections are now more often associated with HSV-1 globally [31]. Although HSV-1 is regularly found during initial infection, HSV-2 is more frequently isolated from this location than HSV-1 at any point because it reacts more frequently from genital tissue than HSV-1 [31]. Increasingly more people are developing genital HSV type 1 infections through oral-to-genital contact. However, reactivations are less prevalent than for HSV type 2 [4]. 

Genital herpes is caused by HSV type 2, which is virtually exclusively sexually transmitted [4,34]. A fraction of persons with genital HSV infection may go undiagnosed or develop severe genital ulcer disease [4].

Primary varicella cases typically have less severe illness than secondary cases in household contacts [8]. Varicella tends to affect elderly persons in the tropics and can result in more severe disease [8]. Deeper pockmarks and more noticeable scars are more common in adults [8].

Infectious virus is present in high quantities in both varicella and zoster skin lesions, which is how they spread to susceptible people [27].

Skin pathogenesis is regulated by innate cellular responses [27]. Strong innate reactions of epidermal and dermal cells control the highly controlled process of lesion development in infected skin xenografts [27]. In the initial phase, infected cells are found around hair follicles, and after that, viral proteins can be identifiedin groups of nearby cells, some of which fuse to form multinucleated polykaryocytes [27]. The cellular transcription factors STAT1 and nuclear factor-B (NF-B), which direct innate immune responses, are upregulated in the uninfected cells that surround infectious foci [27].

### 2.3. Immune Response

Both innate and adaptive immune systems play a role in the immune response to HSV. The fate of an HSV infection is believed to be greatly influenced by the innate antiviral response [35]. As a result, in both mouse models and human investigations, the production of type I interferon (IFN), which is mostly composed of IFN and IFN, has been associated with protection against illness [35]. In vivo, innate immune responses to HSV have also been demonstrated to include various cell types [35]. 

Multiple TLR-binding ligands associated with HSV-1 are known to activate the NF-kB pathway via binding to TLRs, which can induce a resistance of HSV to innate immune responses, particularly TLR signaling and interferon synthesis [1]. HSV infection can increase autophagy, improving how antigens are presented [1]. HSV-1 ICP34.5 gene product, however, counteracts the reaction by interacting with Beclin 1 (Atg6), a crucial component of autophagy [1].

Normal HSV infection results in immunity to stop subsequent infections with the same serotypes but not with the other [16]. A virus needs an infected cell to be alive for as long as feasible and supply the building blocks necessary for reproducing its genetic information and making its protein release considerable volumes of infectious particles from the infected cell [31,36]. Fusogens enable HSV-1 or HSV-2 to fuse its envelope with the host cell membrane during infection [37]. Fusogens are transmembrane fusion proteins with viral encoding that are typically found on the surface of viral envelopes [37]. The viral fusogen gB functions in the case of HSV. The “core fusion machinery” is a multi-protein complex that includes the proteins gB, gD, gH/gL, and their associated receptors; collectively, these proteins carry out the fusion reaction [37].

According to reports, HSVs can either boost cell viability for the production of new virions or encourage the death of cells which can be harmful for their replication and shedding [31]. HSVs, on the other hand, have been shown to cause immune cells to undergo apoptosis [31]. For instance, HSV-1 interacts with Fas/FasL-expressing macrophages infected with the virus and causes natural killer cells (NK cells) to undergo apoptosis, killing dendritic cells [31]. Even though the precise method by which HSVs cause apoptosis in DCs is still unknown, it was discovered that the process was probably mediated by decreased c-FLIP expression since it was susceptible to proteasome-dependent degradation [31].

Several roles of dendritic cells are involved in controlling the immune system’s defenses against infections [38]. Both extracellular and intracellular pattern recognition receptors abundantly expressed by DCs can detect various danger signals, including those connected to infections or malignancies, among other things [38]. DCs can become activated when they come into contact with antigens, producing a variety of cytokines and chemokines and upregulating costimulatory molecules on their surface [38]. After processing these antigens, DCs can go to lymph nodes, exposing CD4+ and CD8+ T lymphocytes to protein-derived peptide fragments [38].

The nuclear factor B (NF-B) pathway can be activated by various methods in HSV-infected cells [39]. The main plasma membrane sensor of HSV contact with antigen-presenting cells is TLR2 [39]. NF-B is activated by HSV infection in a TLR2-dependent manner, and numerous cell types, including macrophages, monocytes, neutrophils, glial and neuronal cells, epithelial cells, and keratinocytes, produce inflammatory cytokines and chemokines [39]. The NF-B pathway is induced by TLR2 activation, which also causes the production of pro-inflammatory cytokines and other cellular proteins in a MyD88-dependent manner [39].

Immune detection of HSV DNA leads to the development of type I IFN and antiviral immunity [39], which is the reason why it was tried as a potential vaccine.

As a result of the disruption of cellular membranes brought on by virus-cell fusion, innate antiviral immunity is also produced. HSV-1 particles cause type I IFN responses without the need to recognize viral nucleic acids [40].

A key component of host adaptive immunity against various intracellular infections and the removal of viruses from the host, CD8+ T cells are immune cells engaged in the response caused by pathogen invasion [41]. Naive CD8+ T cells could develop into Tc1, Tc2, or Tc17 cells after pathogen detection in the context of major histocompatibility complex class I (MHC-I) on antigen-presenting cells (APCs) [41].

Early development of CD8+ T cell responses has been investigated as a way to prevent the establishment of latency due to the critical role that this cell type plays in the defense against HSV [35]. However, this therapeutic approach has shown less promise in animal studies, which determine a latent infection regardless of lesser viral copy numbers in neural sites and reduced viral levels at the site of infection [35]. In reality, several studies show that IFN-producing CD8+ T cells continue to exist in sites of T cell infiltration during neuronal latency, indicating that T cells continue to be stimulated by antigens even after the infection has failed to be cleared [35]. 

In naturally infected individuals, the HSV-2 envelope glycoprotein D (gD2) has been identified as the main target of HSV-2 neutralizing antibodies, with minor contributions from gB2, gC2, and the gH2/gL2 complex [42]. 

The significance of the IFN system in regulating viral replication is shown by the numerous evasion mechanisms that HSV-1 has developed to infect the host and establish latency effectively [43]. Various viral proteins can bloc IFN induction and signaling, or ISG activities [43]. While some of these proteins are freshly generated shortly after the infection starts, others are packed in the teguments of infecting virions and transported to the cytoplasm of infected cells right from the onset of infection [43].

The viral tegument protein kinase US3, which also prevents TNF receptor-associated factor (TRAF) 6, another adaptor of TLR signaling, from being polyubiquitinated, reduces TLR3 expression [44]. Several HSV-1 proteins prevent signaling via the cytoplasmic RLR pathway, which induces IFN, from occurring [44]. An RNA-binding tegument protein called US11 binds to RIG-I and MDA5, preventing them from interacting with MAVS, a necessary common adaptor for starting signaling [44]. This pathway’s crucial component TRAF3 depends on K63-linked polyubiquitination for proper signaling [44]. The biggest tegument protein, HSV-1 UL36, a ubiquitin-specific protease, degrades TRAF3 and blocks its recruitment of TBK1, a protein kinase that phosphorylates IRF3 [44].

HSV-1 can inhibit the activities of numerous interferon-stimulated genes [45]. US11, which binds to both protein kinase R and dsRNA and hinders their direct contact, inhibits the dsRNA-activated kinase protein kinase R [45]. Similar to how it prevents PKR activation by its protein activator, US11 also prevents dsRNA from activating the 2′,5′-oligoadenylate synthetase started by IFN [45]. The tegument protein UL41 inhibits IFN-inducible protein with tetratricopeptide repeats 3’s antiviral activity [45]. The mRNAs of virus inhibitory protein, endoplasmic reticulum-associated IFN-inducible, and zinc finger antiviral protein can all be targeted by UL41, which encourages host mRNA degradation [45].

## 3. Vaccines

Developing a preventive vaccination for herpes simplex virus types 1 and 2 (HSV-1 and 2) is a worldwide public health priority [46,47]. Vaccination is the most effective weapon that humanity holds [48]. 

HSV-1 or HSV-2-caused genital herpes is currently the most prevalent sexually transmitted infection; it causes serious illness in newborns; HSV-1 is the leading cause of infectious blindness in western nations; and prior HSV-2 infection increases the chance of HIV infection by three to six times globally [46,49]. According to estimates, HSV-2 infection is a risk factor for up to 50% of HIV transmissions in sub-Saharan Africa [46,50]. These transmissions are also more likely to happen immediately after the HSV-2 acquisition [46,49,50]. Due to the poor pharmacokinetics of acyclovir/valacyclovir, antiviral treatment for recurrent genital herpes significantly lowers clinical episodes but does not entirely suppress viral shedding or prevent HIV acquisition [51,52]. On the other hand, a preventive HSV vaccination would probably slow the spread of HIV [46]. A part of the most promising HSV vaccine attempts is shown in Table 1.

Given that they are both similarly designed, it is interesting that there is such a noticeable difference between the exceptional efficiency of the recombinant protein vaccine for herpes zoster vaccine and the partial success of the Simplirix vaccine [46]. Understanding the immunological regulatory mechanisms of first genital herpes and natural herpes zoster is key to finding the solution [46]. These include “immunotherapy” vs. prophylaxis—the HSV vaccine aims to control primary infection, whereas the herpes zoster (HZ) vaccine targets disease reactivation—possible variations in the immune responses necessary for control, variations in the mechanism of action of the adjuvants, and variations in the immune evasion mechanisms of each virus [46]. 

The potential therapeutic of HSV vaccination offers a promising substitute for infection-prevention vaccinations [38]. With this approach, symptoms are intended to be avoided without inducing sterilizing immunity [38]. With the use of these vaccines, the latent infection may be controlled, and HSV may become nonpathogenic without the need for infection prevention [38]. Therapeutic vaccinations seek to stop HSV recurrences or lessen the severity and duration of the illness, hence lowering transmission.

In clinical trials, these vaccines have shown promise in reducing the frequency and severity of recurrent HSV infections and the amount of virus shed during periods of asymptomatic shedding. While more research is needed to understand the therapeutic potential of these vaccines fully, they represent a promising avenue for the treatment and prevention of HSV infections.

Several varicella vaccines are used worldwide, with one or two doses recommended in different varicella vaccination programs [46,78]. The shot can be obtained alone or with the measles, mumps, and rubella vaccines (MMRV) [79]. Although varicella vaccinations are approved globally, only a few countries routinely advise varicella immunization as a one- or two-dose regimen [79]. The effectiveness of these vaccines is similar, they are more effective in preventing severe disease than infection, but studies have shown that the second dose improves protection against varicella [46,78].

Although vaccination is essential, unwanted effects can install, such as breakthrough varicella, which can develop in certain people who have received the varicella vaccination after exposure to the wild-type virus [79]. Varicella breakthrough is often lower in intensity and length than varicella in people who have not received the vaccine; clinical signs typically include a rash with less than 50 lesions, the majority of which are maculopapular but can occasionally include vesicular lesions [79]. There have been instances of breakthrough varicella, leading to severe rash, complications, and even death [79].

### 3.1. Subunit Vaccines

Simplirix, a potential GSK vaccine, included just the HSV-2 entrance glycoprotein D (gD) and the adjuvant AS04 [46].

gB, and gD are most frequently used as immunogens in recombinant protein-based subunit HSV vaccines because of their higher targeting of neutralizing antibody production than the other HSV glycoproteins post-infection, [10]. Moreover, they elicit cross-immunity involving both humoral and cellular responses, and they may potentially protect against both HSV-1 and HSV-2 [10]. 

Only HSV1/2 seronegative women with long-term HSV-2-infected partners exhibited 74% effectiveness with Simplirix [80]. Simplirix, however, unexpectedly demonstrated considerable effectiveness against genital herpes caused by HSV-1 (58%) but not HSV-2 (only 20% and minimal efficacy) in the Herpevac study that followed in randomly chosen HSV-1 and 2 seronegative women [80]. Therefore, using recombinant HSV-2 gD, which is largely conserved across the two serotypes, cross-protection against HSV-1 was accomplished [46]. While HSV-2-neutralizing antibody titers were modest, this protection was associated with HSV-1-neutralizing antibody titers [81]. A subsequent effective vaccination response may have been stimulated by subclinical vaginal contact with HSV2 shed by the sick spouse, which would account for the first trial’s superior efficacy [81]. The induction of CD4 Th1 T cells and neutralizing antibodies were both credited with increasing the effectiveness of the new adjuvant dMPL [81]. But no particular CD8 T cells were stimulated [81].

The epitope-based vaccine methods used the recombinant multi-epitope assembly peptide (MEAP) from HSV-2 [55]. These findings showed that MEAP was able to completely protect mice against lethal challenges and induce impressive humoral and cellular immune responses [55].

Results from the scalable GEN-003 vaccination study were comparable to those from earlier clinical studies [56]. Both humoral and cellular immune responses were boosted by GEN-003, which also exhibited a good safety profile [56]. The combination of 60 g antigen/50 g M2 had the greatest impact on virological and clinical measures and calls for further research [56].

Dorsal root ganglia infection and the prevention of chronic recurrent infection were completely protected against by the prophylactic NE01-gD2/gB2 intranasal vaccine [58]. In animals who were already chronically infected, it decreased recurrent lesions and recurrent viral shedding as a therapeutic vaccine [58].

The main advantages of these vaccines could be that they are considered safe for people with weakened immune systems and are easier and cheaper to manufacture than the other types of vaccines. The disadvantages include being less effective at stimulating a strong immune response than other types of vaccines and may require booster shots to maintain immunity.

### 3.2. Live-Attenuated Vaccines

Live attenuated candidates have been avoided during the 60 years of largely unsuccessful attempts to develop an HSV vaccine due to worries about their potential carcinogenicity (at first, HSV-2 was believed to cause cervical cancer) and the possibility that they could recombine with clinical strains to create new, extremely virulent strains [46,82]. Compared to inactivated vaccines, live-attenuated vaccines are more successful in preventing infections and illnesses because they may induce life-long immunity, whereas inactivated and subunit vaccines need repeated doses to maintain protective immunity [10]. Chemical or radiation-based inactivation of virus particles is frequently used as the mechanism of inactivation [33]. When live-attenuated HSV vaccines are administered, genetic changes are made to the virus, preventing it from producing illness in the infected host while inducing a range of immune responses [10]. The problem, however, is that live-attenuated HSV vaccines are challenging to develop since the virus may enter nerve tissue and avoid host immune monitoring [10]. 

Several HSV live-attenuated vaccines have undergone pre-clinical and clinical testing. Although live-attenuated vaccines are particularly good at eliciting a humoral and cell-mediated immune response, vaccine safety is still one of their main drawbacks.

The advantages of such a vaccine could be that it can provide long-lasting immunity and induce a strong and broad immune response. As disadvantages, thesmall risk of causing disease could be mentioned and that they can not be used for people with weakened immune systems.

### 3.3. Naked DNA Vaccines

Genetic vaccines or “naked DNA vaccines” are constructed of plasmid DNA (pDNA), a kind of DNA that is not linked to any other molecules, including proteins and lipids, but is nevertheless intended to express a gene of interest once it enters host cells. There are two methods for employing naked pDNA vaccines against HSV. In the first strategy, cells discovered in the early stages of viral replication are identified and destroyed, including cells previously stimulated to engage immune evasion mechanisms targeted against HSV.

The second strategy uses DNA supplemented with adjuvants or cytokines to enhance the host antibody response to HSV surface glycoproteins gB, gD, or gH/L complex.

The advantages of a DNA vaccine would be that it is safe and easy to manufacture and does not require a live virus, making them safer for people with weakened immune systems.

It is less effective at stimulating a strong immune response than other types of vaccines and may require multiple doses or booster shots to maintain immunity.

### 3.4. Vaccine Vectors

Another unsuccessful attempt was to use glycoproteins C (gC2) and D (gD2) to stimulate humoral immunity and UL19 (capsid protein VP5) plus UL47 (tegument protein VP13/14) as T cell immunogens [76]. The HSV-2 gC2 and gD2 proteins were expressed in baculovirus, while the UL19 and UL47 genes were expressed from replication-defective adenovirus vectors [76]. Considerable efforts should be made to increase the effectiveness of this type of vaccine [76]. A strategy is to increase the amount of B cell immunogens by incorporating different entry molecules such as glycoproteins B, H, and L or by inserting glycoprotein E, an additional immune evasion molecule [76]. Another strategy is to increase the amount of T cell immunogens present, such as UL39 (ribonucleotide reductase subunit 1), UL1 (glycoprotein L), and immediate early proteins ICP4 and ICP0, which are all important T cell targets in HSV-2-exposed, antibody-seronegative individuals [76]. Examining new adjuvants that can improve the strength and longevity of immune responses is a third factor to consider [76].

The advantages of vaccine vectors would be that it can induce a strong and broad immune response and it is safe and well-tolerated. However, at the same time it is relatively new and still undergoing testing, so long-term safety and effectiveness are not yet fully understood. They may require multiple doses or booster shots to maintain immunity.

### 3.5. Replication-Defective Viruses as Vaccines

Replication-defective vaccines are made by deleting a crucial gene in a mutant virus, rendering it unable to perform the normal viral tasks necessary for replication [10].

These vaccines were tried without success for human immunodeficiency virus and herpes simplex virus when the classical viral vaccines, such as an inactivated virus or live-attenuated virus vaccine, were not working [83]. These vaccines can be safe and, at the same time, express viral antigens inside infected cells because replication-defective viruses serve both as vaccines and vectors for the expression of heterologous antigens [83].

Such a technique was tried on HSV vaccines in the early 80 s when cotransfection of cells with helper virus DNA and chimeric repeat units of bacterial plasmid DNA were performed to generate defective genomes composed of reiteration of the seed HSV-pKC7 repeats [84]. 

Later in 1994, replication defective HSV-1 virus was used as a vaccine, the resulting mutant viruses being able to infect normal cells and express early and late proteins without completing the replication [85].

A highly studied vaccine candidate virus, the dl5-29 generated by the deletion of two HSV-2 essential for replication genes, UL5 and UL29, emerged in 1999, initially with many promises since the newly created virus’s defect was the lack of latency [86].

A HSV-2 gH mutant virus was also studied because it was limited to a single replication cycle, but unfortunately, it generated latent infection in the inoculated laboratory animals tested [87].

### 3.6. Trivalent Subunit Vaccines

Today’s vaccinations may generally be divided into two categories: live and killed [88]. In general, killed or attenuated viral vaccines, such as whole virus and subunit viral vaccines that have been killed, are assumed to be safer [89]. In contrast, live attenuated viral vaccines are expected to offer more enduring and long-lasting protection [89].

Unfortunately, at the moment, there are no approved herpes vaccines, and recent clinical trials of subunit vaccines failed to achieve endpoint goals [88].

An older yet hardly studied herpes subunit vaccine, gD2, provided similar protection with a live attenuated herpes virus vaccine, VC2, but with lower protection duration [90,91].

One of the most popular and promising vaccine type against HSV-1 and HSV-2 remains the subunit vaccine [10]. Additional advancements in these vaccine investigations have occurred recently [10]. The trivalent vaccine containing herpes simplex virus type 2 (HSV-2) glycoproteins C, D, and E (gC2, gD2, gE2) produced in baculovirus and administered with CpG/alum as adjuvants protected guinea pigs against HSV-2 vaginal infection and proved to cross-protect against HSV-1 [92].

### 3.7. Nucleoside-Modified mRNA Vaccines

Recently, nucleoside-modified mRNA vaccines have shown great promise for developing vaccinations against infectious diseases [10]. According to valid data, they may perform better than conventional subunit vaccinations [74]. 

The vaccine field had changed since the COVID-19 pandemic when two nucleoside-modified mRNA vaccines emerged. This technology has many advantages, such as accelerated immunogen discovery, induction of robust immune responses, and rapid scale-up of manufacturing [93]. It is well known that for 8 decades, there was no success in developing genital herpes vaccines [93]. The advent of mRNA technology can potentially change this [93]. 

Trivalent nucleoside-modified mRNA vaccines in lipid nanoparticles are being investigated for their effectiveness in protecting against HSV-1 and HSV-2, similar to trivalent subunit vaccinations [74]. The translation is improved, and inflammatory adverse effects are decreased by mRNA modifications [10].

### 3.8. Multivalent DNA Vaccines

HSV potential vaccine candidates against genital herpes infection were studied on animal models such as mice, rats, and guinea pigs using the same evaluation criteria used in human trials [89,94]. 

The multivalent vaccines contain more than one antigen in order to induce a better immune response [95]. By lowering the clinical signs of infection and causing efficient viral eradication in a mouse model, the recently reported, multivalent DNA vaccine, SL-V20, was able to provide a new and effective vaccination against vaginal HSV-2 [95].

### 3.9. Next-Generation Live-Attenuated Virus Vaccines

In a study on guinea pig, when the HSV-2 challenge was performed during the height of the immune responses, 3 weeks following the third immunization, the live attenuated vaccine, VC2, offered superior protection compared to the subunit gD2 MPL/Alum vaccine [89]. In the guinea pig model of genital HSV-2, the live-attenuated prophylactic HSV vaccination, R2, proved productive, particularly through the ID route [72]. 

Compared to gD2, VC2 offered better defense against acute illness, acute virus replication, virus levels in brain tissue, recurrent illness, and recurrent virus shedding [89]. A live-attenuated vaccine that reproduces in non-neural tissue but is ablated for transmission into the nervous system may induce protective immune reactions without resulting in neurological problems or creating life-long infections [72]. Acute vaginal replication was decreased, brain tissues were protected, and probably most significantly, recurrent illness and recurrent viral shedding, two goals that can only be studied in the guinea pig model, were different [79]. The disparities between the two vaccines were much more pronounced when comparing the protection conferred six months after inoculation [79]. A potential strategy for HSV vaccines is using live-attenuated HSV vaccines that can successfully increase mucosal tissues but are ablated to prevent neuroinvasion [72].

The ID route in the VC2 proved to be more effective than the IM route of administration [88]. 

### 3.10. gD2 Deletion Vaccine

The main strategy for HSV prevention has been concentrated on subunit protein vaccines that lack the HSV-2 envelope glycoprotein D and has targeted genital illness [96]. A group of researchers employed a completely different strategy and created a single-cycle candidate HSV-2 vaccine strain known as gD-2 by deleting the gene encoding gD-2, which is necessary for viral entrance and cell-to-cell transmission [96].

Two potential recombinant viral protein vaccine candidates underwent testing at the end of the last century [97]. HSV2 entrance glycoproteins B and D in combination with the adjuvant MF59 from an oil-in-water emulsion made up of the Chiron vaccine candidate [97]. When given to patients with recurrent genital herpes, the medication produced large levels of neutralizing antibodies but had no long-lasting or appreciable impact on the frequency of outbreaks [97].

### 3.11. Future Perspectives

Reverse vaccinology is a widely used approach that screens whole viral genomes and identifies genes that may provide potent epitopes for improved vaccines [98]. Before recommending a peptide segment, it is advised to make the following analyses: epitope predictions, epitope accessibility predictions, and 3D structure accessibility [98]. Synthesis of the theoretically determined peptide sequences is challenging when bioinformatic epitope identification is finished [98]. To guarantee the correct immunological response is evoked and that the protein does not fold in vivo in a way that would interfere with its expected antibody-antigen binding site, these peptides must be produced and evaluated in animal models [98].

Bioinformatic approaches incorporate all of these elements as part of the in silico design of a candidate, just as other approaches to vaccine development take into account the genomics of a specific virus, host immune response, and components that enhance the desired effects or reduce adverse effects [10,89].

Type I membrane protein programmed death ligand-1 (PD-1) was first discovered as a gene involved in programmed cell death [99]. PD-1, a member of the immunoglobulin superfamily, is expressed as a monomer on activated T cells, B cells, NK cells, dendritic cells, and monocytes. PD-1 and PD-L1 trigger the SHP2 signaling pathway [99]. SHP2 inhibits T cell activation by downregulating T cell receptor pathway signaling molecules such as Zeta-associated protein of 70 kDa, phosphatidylinositol 3-kinase, and phospholipase Cγ2 [99,100]. The PD-1/PD-L1 checkpoint fine-tunes immunity, which helps the host survive because an overactive immune response poses health risks [99,100].

The PD-1/PD-L1 pathway ameliorates the illness by limiting the immune response, and the symptoms of herpetic stromal keratitis are worse when the immune response is robust [101]. During persistent viral infections, inhibiting programmed death ligand-1 (PDL-1) has been demonstrated to improve virus-specific CD8 T cell activity [102]. Yet, according to some findings, HSV infection upregulates PD-1/PD-L1, and elevated PD-1/PD-L1 leads to cell-mediated immunity becoming exhausted, increasing the viral copy number [101]. For instance, in a mouse model of latent infection, blocking PD-L1 with a monoclonal antibody restored CD8+ T cell activity in the ganglia [101].

The primary and secondary CD8 T cell responses to acute viral infection are improved by blocking the PDL-1 interaction during priming [102]. This shows that enhancing the epitope-specific T-cell response in a preventive vaccination system may be accomplished by targeting the PD-1: PDL-1 interaction in conjunction with other strategies [102].

To avoid HSV reactivations from neurons of the dorsal root ganglia and to lessen the severity of inflammatory lesions in the genital tract, HSV-specific CD8+ T lymphocytes are essential [87]. The long-term objective is to create a genital herpes immunotherapeutic vaccine [87].

Combinatorial vaccination methods appear to be the most suited in the current situation to develop HSV vaccine studies because most of the current HSV vaccine candidates were not individually promising in clinical trials [87]. Practical difficulties with combinatorial applications necessitate optimization using animal models [87].

Creating a vaccine for HSV-2, which can last a lifetime, would significantly improve sexual and reproductive health worldwide [103]. A prophylactic vaccine with only 75% efficacy against infection could reduce HSV-2 incidence by more than 55% after 10 years, given 50% vaccination coverage of 14-year-olds and catch-up vaccination through age 29. It could also prevent about 10% of incident HIV infections over the same period, according to one study that modeled both HSV-2 and HIV transmission in sub-Saharan Africa [104].

Therapeutic vaccinations are a new idea in public health that has received little modeling research to date; further study is necessary to fully understand their projected advantages for the individual and population and who and how they should be targeted for the best use of available resources [104]. Therapeutic vaccinations might be used solely to lessen symptoms in people who have symptoms of infection [104]. Depending on how it affects the transmission, this kind of therapeutic vaccination program might only benefit a few people. Still, it could significantly enhance the quality of life for those who have been diagnosed with genital herpes [104].

Although a partially effective prophylactic vaccination may still be helpful if it lowers the threshold for infection or prevents or lessens sickness, an ideal prophylactic vaccine would prevent infection [104]. By avoiding infection or decreasing the infectivity of an HSV2-infected person through decreases in shedding or clinical recurrences, these vaccinations may lower the incidence of HSV2 [104]. In contrast, vaccinations might raise the risk of HSV2 if they decreased illness symptoms but did not impact viral shedding [104].

Early adolescent prophylactic HSV-2 vaccinations would need to be successful in those who are HSV-1-seropositive [105]. This is because HSV-1 is acquired throughout infancy, and in many low-middle income countries, seroprevalence is high by the start of adolescence [105]. In several high-income countries, the typical age at which people become newly infected with HSV-1 is rising [105]. It would be necessary to deliver the vaccine early in life to protect against both HSV types [105]. Larger clinical trials will likely be needed for the development of preventative vaccinations than for therapeutic vaccines [105].

## 4. Conclusions

Although HSV and VZV share many structural similarities, there is no available vaccine for the herpes simplex virus, a situation given most probably by their diverse pathogenicity, the immune response they elicit during infection, and latency. There are multiple aspects to consider when designing a new vaccine, mainly immunogenicity, route of administration, and potential adjuvants. Vaccines offer a high protectivity for varicella, but there are still cases of disease even after vaccination. Numerous types of vaccines have been tried for HSV, from replication-defective viruses as vaccines, naked DNA vaccines, live-attenuated vaccines, trivalent subunit vaccines, nucleoside-modified mRNA nanoparticle vaccines, gD2 deletion vaccines, but their efficacy proved to be reduced. The main reasons for their reduced efficacy were the possibility of incomplete protection, which may occur due to individual differences in immune response or the herpes virus’s ability to evade the immune system and the timing of vaccination. Another setback of the efficacy of herpes vaccines is the high frequency of seropositive people and the adjuvant used. Newer alternatives are studied, such as bioinformatic approaches, combinatorial vaccination methods, or the help of programmed death ligands inhibitors. 

## Figures and Tables

**Table 1:** HSV vaccine studies.

Types of Vaccine	Study	Mechanism of Action	Advantages and Disadvantages	References
Subunit vaccine	CTL, HTL and BCL epitopes	Production of neutralizing antibodies and some can block the immune evasion [18]	Advantage: − greater vaccine safety − no risk of infection Disadvantage: − they elicit a reduced immune response compared with the live attenuated vaccines − may need several doses	[53]
	MEAP			[54]
	GEN-003/MM-2 − gD2 truncated + ICP4 fragment (29.2 kD) + Matrix M2 Adjuvant			[55]
	Chiron glycoproteins gB and gD			[56]
	GlaxoSmithKline—purified carboxy-terminal truncated gD2 expressed in CHO cells			[57]
	Bivalent HSV-2 Subunit (gD2 and gB2) + Nanoemulsion adjuvant NE01			[58]
	CD8+ T cell peptide epitopes (UL44 aa400–408, UL9 aa196–204, and UL25 aa572–580)			[59]
	G103—three recombinantly expressed HSV-2 proteins (gD and the UL19 and UL25 gene products) adjuvanted with synthetic TLR4 agonist glucopyranosyl lipid A (GLA) formulated in stable emulsion			[60]
	32 synthetic 35mer HSV-2 peptides non-covalently complexed with recombinant human Hsc70 protein (named HerpV, formerly AG-707)			[61]
	intranasal trivalent HSV-2 subunit vaccine (gC2, gD2, and gE2)/alum adjuvant			[62]
Live-attenuated Vaccines	R7017 and R7020	They stimulate both humoral and cell-mediated adaptive immune responses as well as innate immunity; can lead to a impairment of virus replication	Advantages: − evoke a longer-lived immune response − some could be efficient as both prophylactic and therapeutic vaccines Disadvantages: − concerns about vaccine safety − concerns about neurotropism, transmission, or reactivation at a later time	[63]
	herpes simplex virus gamma 1 34.5 deletion mutants			[64]
	ICP0(−) mutant virus (HSV-2 0DeltaNLS)			[65]
	RAV 9395			[66]
	a vaccine lacking both copies of γ134.5 as well as UL55, UL56 and the US10-12 region of HSV-2			[67]
	live-attenuated HSV-1 VC2 vaccine strain			[68]
	replication-competent controlled HSV-1 vectors (HSV-GS3 and HSV-GS7)			[69]
	HSV-2 with US6 (gD) deletion			[70]
	replication-defective HSV-2 dl5-29			[71]
	R2 non-neuroinvasive HSV-1 vaccine (HSV1-GS6264, 5 missense mutations in UL37)			[72]
DNA vaccines	naked plasmid DNA (pDNA) vaccine expressing herpes simplex virus type 1 (HSV-1) glycoprotein B (gB)	Production of neutralizing antibodies, sIgA and increased activity of natural killer cells and splenocytes [18]	Advantage: − no risk of infection − targeted immune response − stability − well tolerated Disadvantage: − lack of substantive data in humans − at the moment, expensive to produce	[73]
	nucleoside-modified mRNA encoding gC2, gD2, and gE2 + lipid nanoparticles			[74]
	Vaxfectin-adjuvanted plasmid DNA vaccine			[75]
Viral vector agents	herpes simplex virus 2 (HSV-2) glycoproteins C (gC2) and D (gD2) and UL19 (capsid protein VP5) and UL47 (tegument protein VP13/14) expressed in baculovirus	The delivery of one or more antigens encoded in the context of an unrelated, modified virus induces immune responses against the respective target pathogen	Advantage: − highly immunogenic due to the vector’s natural MAMPs − the immune response is both cell-mediated and humoral Disadvantage: − they genetically modified organisms and are there is a potential risk to human health and environment associated with the release of these organisms − potential integration into the host genome or too high or persistent replication	[76]
	lentiviral vector-based HSV-1 glycoprotein B vaccine			[77]

## Data Availability

No new data were created or analyzed in this study. Data sharing is not applicable to this article.
